# Increasing Burden of Nursing Care on the Treatment of COVID-19 Patients in the Aging Society: Analyses During the First to the Third Wave of Pandemic in Kyoto City, Japan

**DOI:** 10.3389/fmed.2021.767110

**Published:** 2021-11-18

**Authors:** Kohei Fujita, Eriko Kashihara, Osamu Kanai, Hiroaki Hata, Akihiro Yasoda, Takao Odagaki, Tadashi Mio

**Affiliations:** ^1^Department of Infectious Diseases, National Hospital Organization Kyoto Medical Center, Kyoto, Japan; ^2^Division of Respiratory Medicine, Center for Respiratory Diseases, National Hospital Organization Kyoto Medical Center, Kyoto, Japan; ^3^Department of General Medicine, Tenri Hospital, Nara, Japan; ^4^Department of Surgery, National Hospital Organization Kyoto Medical Center, Kyoto, Japan; ^5^COVID-19 Response Headquarters, National Hospital Organization Kyoto Medical Center, Kyoto, Japan; ^6^Division of Endocrinology, Metabolism and Hypertension Research, Clinical Research Institute, National Hospital Organization Kyoto Medical Center, Kyoto, Japan

**Keywords:** COVID-19, aging society, nursing care, elderly patients, healthcare workers

## Abstract

**Background:** The coronavirus disease 2019 (COVID-19) pandemic is associated with a heavy burden on patient's mental and physical health, regional healthcare resources, and global economic activity. An aging society such as Japan has many retirement homes and long-term stay hospitals for the elderly and their inhabitants. During the COVID-19 pandemic, disease clusters are often identified in retirement homes and long-term stay hospitals. Although we hypothesize that additional burdens of nursing care for elderly patients will reinforce the anxiety and exhaustion of medical staff and healthcare resources in the aging society, the actual situation is not well understood. In this study, we aimed to evaluate the current situation and countermeasures of the COVID-19 pandemic in the aging society.

**Methods:** We reviewed COVID-19 patients who required hospitalization at the National Hospital Organization Kyoto Medical Center, a 600-bed capacity hospital located in Kyoto, Japan, between 1 April 2020 and 31 March 2021. We assessed the characteristics of the COVID-19 patients, disease severity, duration of hospitalization, outcome at discharge, degree of activities of daily living (ADLs), and complications unique to elderly patients.

**Results:** We enrolled 118 patients who required hospitalization during the study period. Approximately 40% of the patients were aged ≥ 80 years. Dementia (27.1%) was the most prevalent underlying disease, followed by diabetes mellitus (23.7%) and chronic kidney disease (23.7%). Approximately 60% of hospitalized COVID-19 patients had impaired ADL at admission. The COVID-19 patients aged 80 years or older required significantly more longer-term hospitalization than the COVID-19 patients aged under 80 years (15.5 ± 8.2 vs. 13.1 ± 7.7, *P* = 0.032). In elderly patients aged 80 years or older, approximately 50% of patients had geriatric mental disorders, and approximately 70% had bedridden status and feeding difficulty. Poor ADL at admission was significantly associated with COVID-19 mortality (Odds ratio, 5.6; 95% confidence interval, 1.04–45.2; *p*-value = 0.044).

**Conclusions:** The proportion of elderly patients aged 80 years or older was relatively high during the hospitalization for COVID-19. Poor ADL at admission in these elderly patients was significantly associated with poor prognosis of COVID-19. We should keep in mind that healthcare workers are forced to have an additional burden of nursing care in the aging society during the COVID-19 pandemic. Therefore, interventions to reduce the burden are urgently required.

## Background

Coronavirus disease 2019 (COVID-19) is caused by severe acute respiratory syndrome coronavirus-2 (SARS-CoV-2). COVID-19 was first identified in Wuhan, China, in December 2019. The outbreak of COVID-19 had rapidly spread worldwide, and the World Health Organization declared a pandemic on 11 March 2020 (1). The clinical course of COVID-19 is mild and self-limiting upper respiratory infection symptoms in young healthy subjects; however, severe respiratory failure is often observed in elderly patients with chronic diseases (2, 3). The COVID-19 pandemic is associated with a heavy burden on patient's mental and physical health, regional healthcare resources, and global economic activity. As is well known, Japan is facing the most aging society worldwide (4). There are many retirement homes and long-term stay hospitals for the elderly and their inhabitants in Japan. During the COVID-19 pandemic, disease clusters are often identified in retirement homes and long-term stay hospitals (5). Elderly patients with chronic diseases had been mainly affected by the COVID-19 pandemic between the first and third waves of the pandemic in Japan. A recent study has shown that clinicians and clinical teams for COVID-19 patient care had struggled with the complexity of providing high-quality care because of the following difficulties: crisis capacity, resource limitation, and multiple unprecedented barriers to care delivery (6, 7). In addition, healthcare workers had a higher prevalence of stress, anxiety, depression, and psychological distress than the general population during the COVID-19 pandemic (8).

In the course of COVID-19 treatment, we noticed that there are many more elderly people than expected, and that the burden of care is much greater. Therefore, in this study, we aimed to investigate the fact that in aging cities and countries where there are many elderly people, the burden of nursing care, in addition to the treatment of infectious diseases themselves, is high for medical staff and healthcare resources.

## Patients and Methods

We reviewed COVID-19 patients who required hospitalization at the National Hospital Organization Kyoto Medical Center, a 600-bed capacity hospital located in Kyoto, Japan, between 1 April 2020 and 31 March 2021. Our institution is an inpatient-only medical facility for COVID-19 and does not include outpatients for COVID-19. All COVID-19 patients who were admitted to the hospital are included. During this period, Japan experienced several major outbreaks of COVID-19: the first, second, and third waves of the pandemic. During this pandemic, most tertiary medical centres or hospitals in Kyoto City have accepted mild to severe COVID-19 patients. All COVID-19 patients admitted to our hospital were diagnosed by SARS-CoV-2 PCR tests. Because COVID-19 was designated as a specific infectious disease in Japan, all COVID-19 patients were hospitalized according to the recommendation of public authorities and treated by publicly funded health care. To avoid this concentration, COVID-19 patients living in Kyoto City were assigned to suitable hospitals by the public authorities. The population aging rate of Kyoto City increased to 28.0% in 2019, indicating that 1 out of 3.5 people living in Kyoto City was an elderly aged 65 years or older (9). We assessed the characteristics of COVID-19 patients, disease severity, duration of hospitalization, outcome at discharge, degree of activities of daily living (ADLs), and complications unique to elderly patients. Disease severity was defined as mild, moderate, or severe according to the Japanese COVID-19 treatment guidelines published by the Japanese Ministry of Health, Labour and Welfare (5). COVID-19 Registry Japan (COVIREGI-JP) of National Center for Global Health and Medicine were used for this study with permission. The study data were collected and managed using REDCap (Research Electronic Data Capture) a secure, web-based data capture application hosted at JCRAC data center of National Center for Global Health and Medicine.

## Statistics Analyses

Statistical analyses were performed using the Statistical Package for the Social Sciences (SPSS) version 26 (International Business Machines Corporation SPSS, Chicago, IL, USA). The Wilcoxon rank-sum test was used to compare the duration of hospitalization and age-specific mortality rate between the two groups. The Kruskal-Wallis test was used to compare the mortality rate in different ADLs groups. Logistic regression analysis was used in the multivariate analysis for the factors associated with mortality in the COVID-19 patients.

## Results

**Figure 1:**
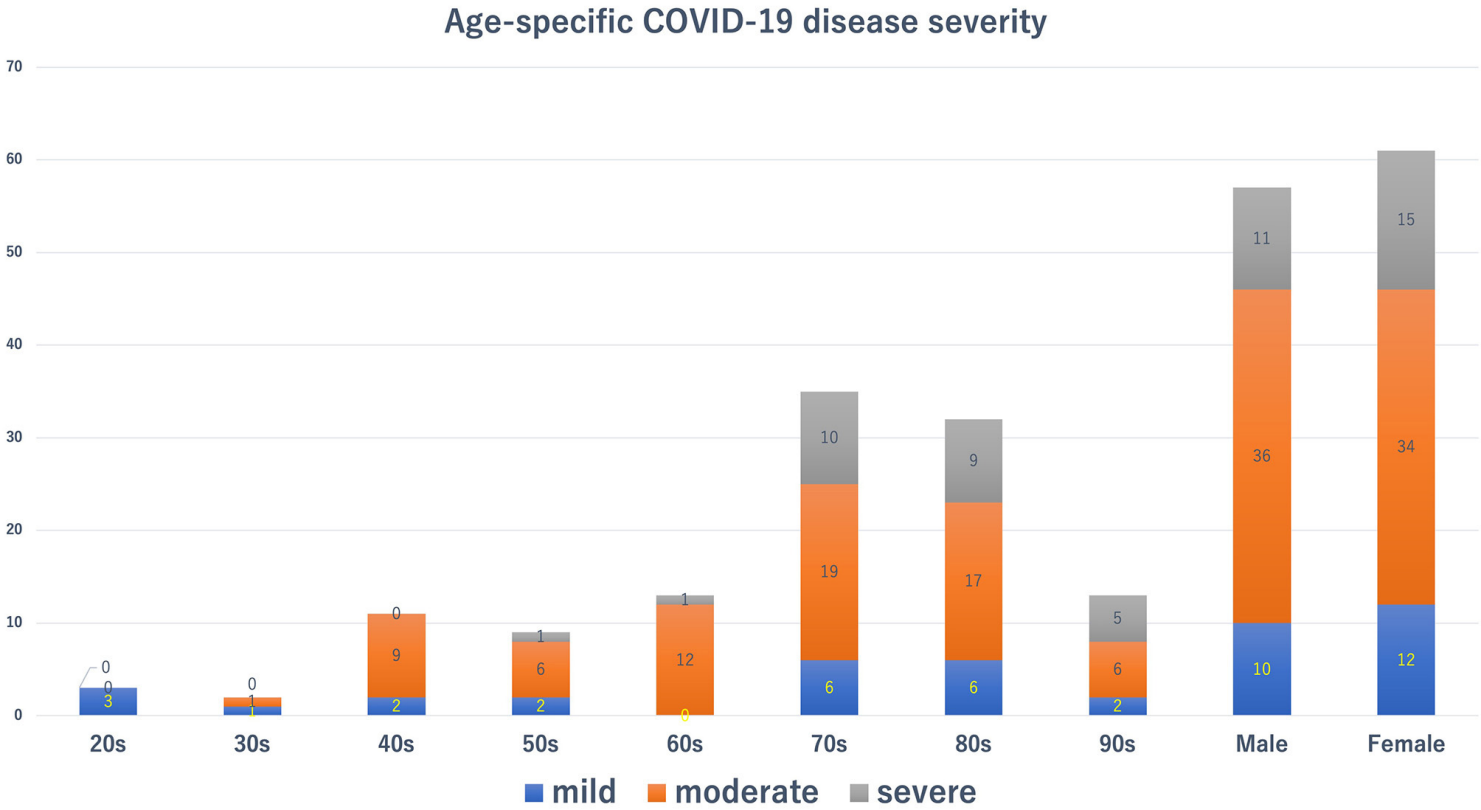
Age-specific coronavirus disease 2019 disease severity. Blue, red, and gray bars show mild, moderate, and severe disease status, respectively.

**Figure 2:**
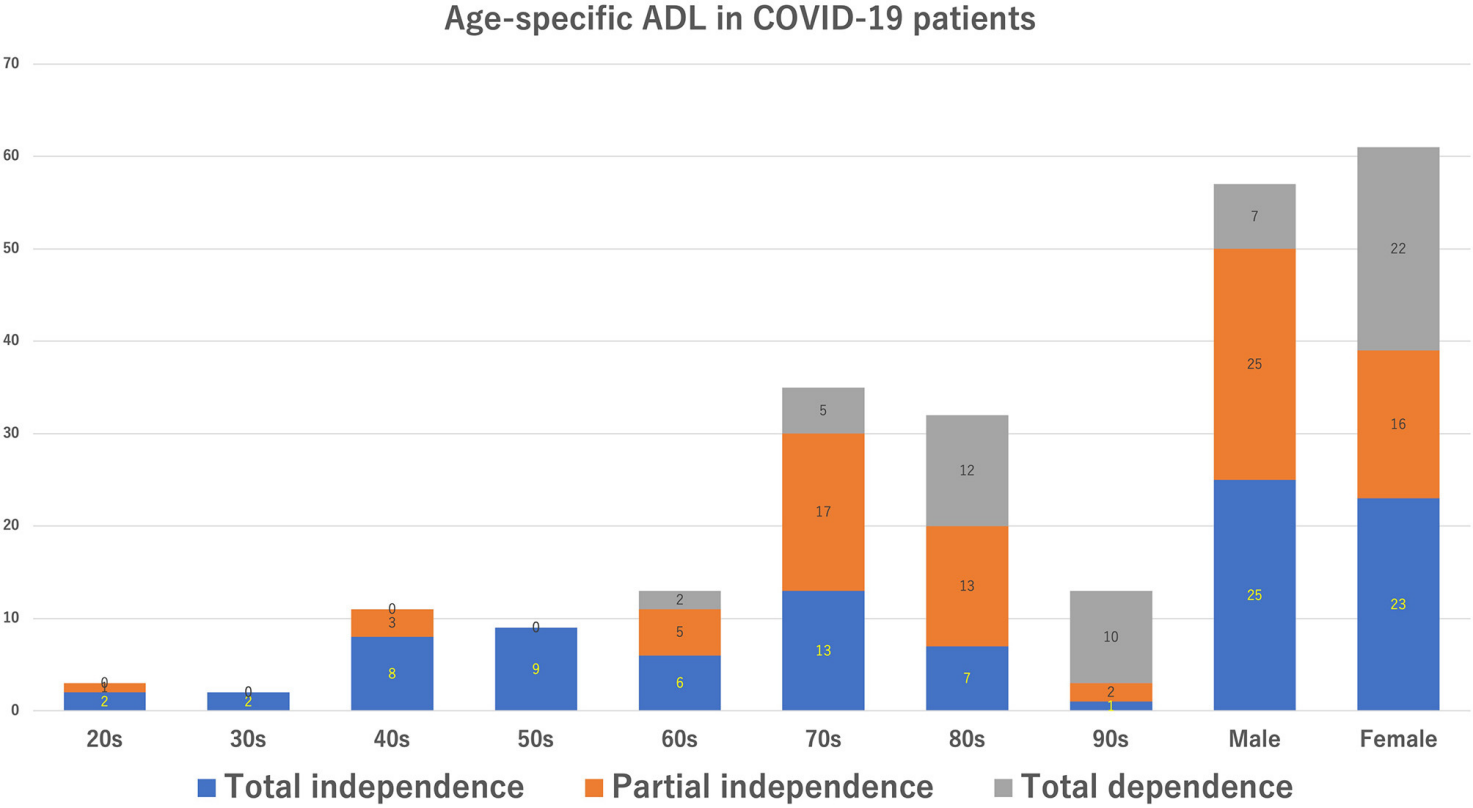
Age-specific activities of daily livings in the coronavirus disease 2019 disease patients.

We enrolled 118 patients who required hospitalization during the study period. The characteristics of COVID-19 patients hospitalized in our hospital are shown in Table 1. Patient's mean age was 71.3 ± 17.4 years. The female-to-male ratio was roughly half and half. Approximately 40% of the patients were aged ≥ 80 years. Dementia (27.1%) was the most prevalent underlying disease, followed by diabetes mellitus (23.7%) and chronic kidney disease (23.7%). Figure 1 shows age-specific COVID-19 disease severity. Hospitalization was most commonly needed in the elderly in their 70's, followed by the 80 and 60's. Most patients in their 20 and 30's had mild disease, whereas the majority of patients in their 40, 50, and 60's had moderate disease. Furthermore, the vast majority ( ≤ 80%) of patients aged ≥ 70 years had moderate and severe diseases. The ADLs of the COVID-19 patients are shown in Table 1, Figure 2. Approximately 60% of hospitalized COVID-19 patients had impaired ADLs at admission. As the patient's age group increased, the ADL at the time of admission decreased. The decrease of ADL was more prevalent in female patients.

**Table 1:** Characteristics of the coronavirus disease 2019 patients.

**Characteristics**	***N*** **= 118**
Age, years	71.3 ± 17.4
Sex (female)	61 (51.7)
Elderly patients (aged 80 years ≤)	45 (38.1)
**Underlying diseases**	
Cardiovascular diseases	22 (18.6)
Cerebrovascular diseases	12 (10.2)
Dementia	32 (27.1)
Diabetes mellitus	28 (23.7)
Chronic pulmonary disease	17 (14.4)
Chronic kidney disease	28 (23.7)
Malignant disease	13 (11.0)
Use of immunosuppressants	8 (6.8)
Use of biologic agents	1 (0.85)
Duration of hospitalization, days	14.1 ± 8.0
**Activities of daily livings**	
Total independence	48 (40.7)
Partial independence	41 (34.7)
Total dependence	29 (24.6)
Disease severity	
Mild	22 (18.6)
Moderate	70 (59.3)
Severe	26 (22.0)

*Data are shown as mean ± standard deviation or number (%)*.

**Figure 3:**
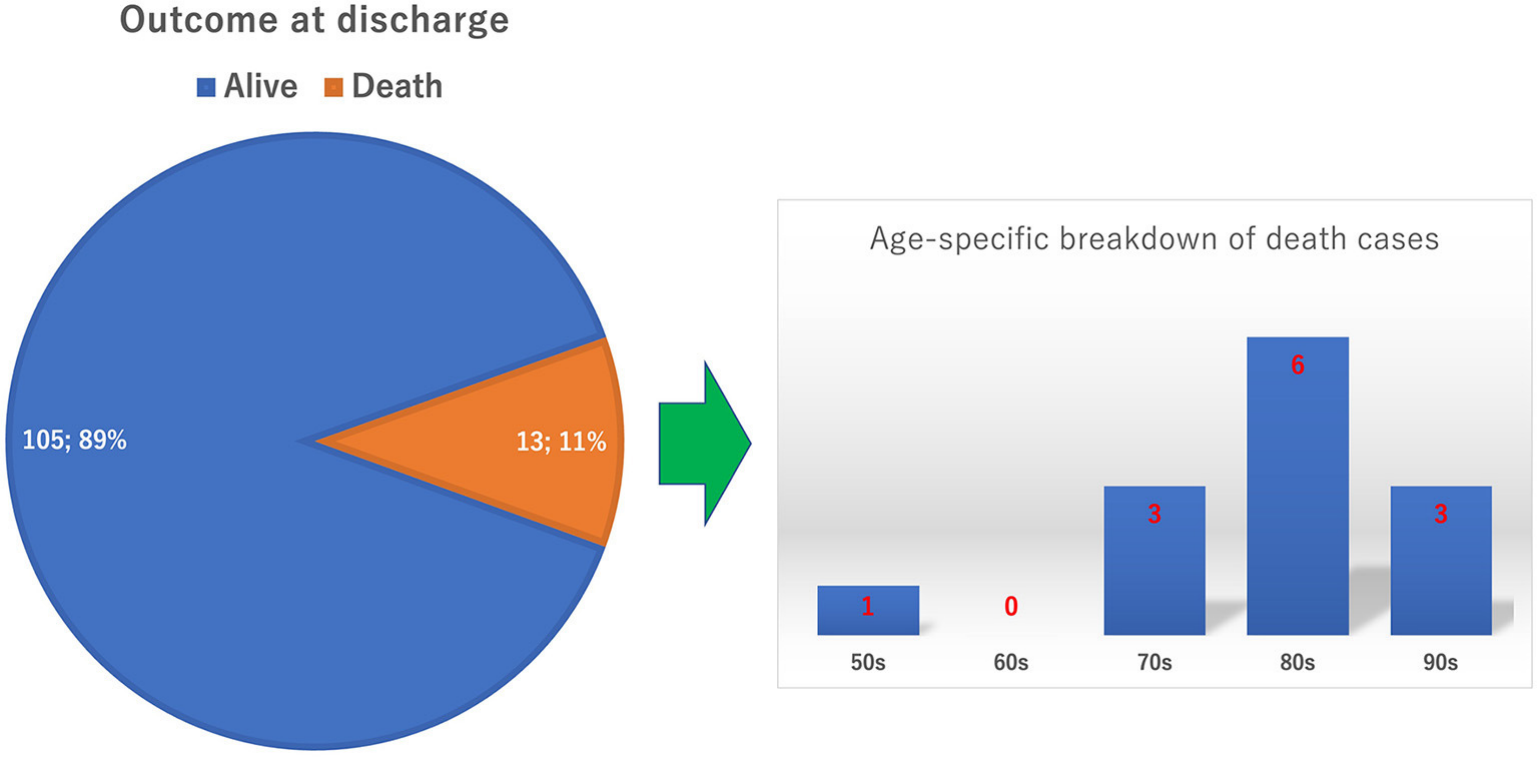
Outcome at discharge. The circle graph shows the percentages of alive and death cases. The bar graph shows the age-specific breakdown of the death cases.

**Figure 4:**
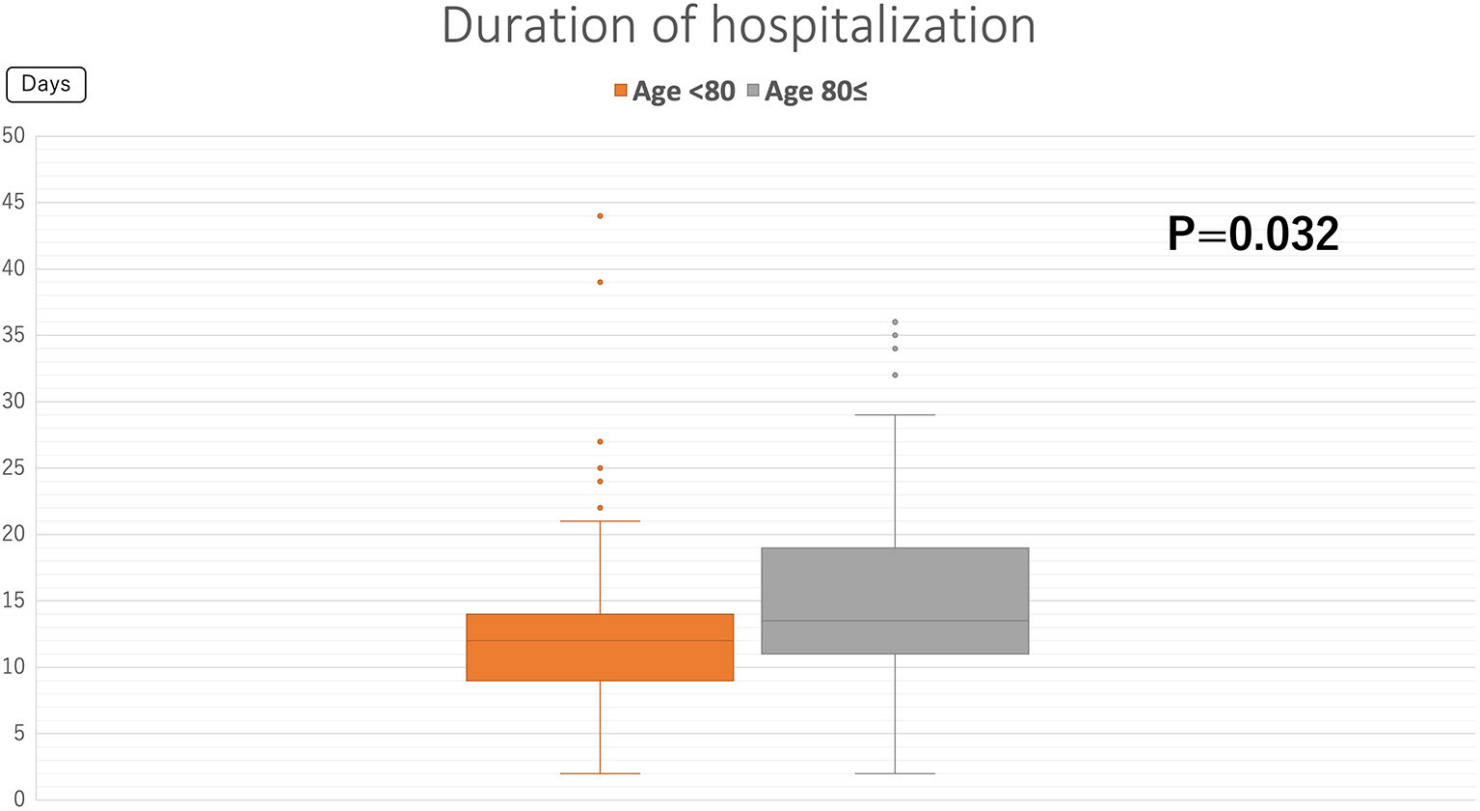
Duration of the hospitalization. Orange and gray box plots show the mean duration of hospitalization in patients aged < 80 years and those aged ≥ 80 years, respectively.

Figure 3 shows the outcomes of COVID-19 patients at discharge. The COVID-19 mortality rate at our hospital was 11%. All but one patient was in their 70 to 90's. Figure 4 shows the duration of the hospitalization. COVID-19 patients aged 80 years or older required significantly more longer-term hospitalization than the COVID-19 patients aged under 80 years (15.5 ± 8.2 vs. 13.1 ± 7.7, *P* = 0.032). Table 2 shows the complications unique to elderly patients in the COVID-19 care unit. In all patients, approximately 40% had a bedridden status and feeding difficulty. For elderly patients aged 80 years or older, approximately 50% of patients had geriatric mental disorder, and ~70% had bedridden status and feeding difficulty. Table 3 shows the mortality rate in each ADL and age-group. Univariate analysis shows that the mortality rate is significantly higher in the group of elderly patients aged at 80 or over (*p* = 0.0303) and in the group with poor ADL (*p* = 0.0041). Table 4 shows the results of multivariate analysis. Poor ADL is significantly associated with COVID-19 mortality (Odds ratio, 5.6; 95% confidence interval, 1.04–45.2; *p*-value = 0.044).

**Table 2:** Complications unique to elderly patients.

**Complications**	**All patients**	**Patients aged 80** **years or older**
	***N*** **= 118**	***N*** **= 45**
Bedridden status	46 (39.0)	31 (68.9)
Feeding difficulty	52 (44.1)	34 (75.6)
Enteral alimentation	19 (16.1)	11 (24.4)
Fluid replacement	33 (28.0)	23 (51.1)
Disquiet/delirium	38 (32.2)	26 (57.8)
Dementia/communication difficulty	40 (33.9)	24 (53.3)
Need continuous sputum suction	27 (22.9)	18 (40.0)

*Data are shown as number (%)*.

**Table 3:** Mortality in each activities of daily livings and age group.

	**Number** **of patients**	**Number** **of deaths**	***p*** **-value**
	***n*** **= 118**	***n*** **= 13**	
**Activities of daily livings**			
Total independence	48 (40.7)	2 (15.4)	0.0041
Partial independence	41 (34.7)	3 (23.1)	
Total dependence	29 (24.6)	8 (61.5)	
**Age group**			
aged 80 years ≤	45 (38.1)	9 (69.2)	0.0303
Aged 80 years>	73 (61.9)	4 (30.8)	

*Data are shown as number (%)*.

**Table 4:** Multivariate analysis for the factors associated with mortality in the coronavirus disease 2019 patients.

**Variables**	**Odds ratio (95% CI)**	***P*** **-value**
Poor ADL (Total dependence)	5.6 (1.04–45.2)	0.044
Older age (aged 80 years ≤)	2.15 (0.53–9.69)	0.28

*ADL, activities of daily livings; CI, confidence interval*.

## Discussion

In our hospital, almost 40% of hospitalized COVID-19 patients were aged 80 years or older. We found that 30 to 40% of COVID-19 patients in our hospital experienced complications unique to elderly patients. According to the Japanese government statement, elderly patients aged 80 years or older accounted for 10 to 15% of hospitalized COVID-19 patients in Japan (5). Our hospital had accepted approximately three times higher percentage of elderly COVID-19 patients than the average of other hospitals in Japan. This might be caused by the COVID-19 clusters that occurred in nearby retirement homes and long-term stay hospitals.

Notably, we revealed that only 40% of the COVID-19 patients in our hospital could maintain their ADLs of total independence at admission. The remaining 60% of the patients had impaired ADLs. Approximately 30–40% of all hospitalized patients have some forms of complications unique to elderly patients. In patients aged 80 years or older, the rate of these complications increased by 50 to 70%. Furthermore, this study showed that poor ADL at admission was significantly associated with high mortality. These facts clearly indicate that healthcare workers belonging to COVID-19 care units had a considerable burden of nursing care from the early stages of hospitalization. Furthermore, because our study revealed that elderly patients aged 80 years or older had a significantly longer duration of hospitalization, this situation would exacerbate the bed turnover rate and result in additional physical and mental burden in healthcare workers. Mehta et al. have reported that many healthcare workers are concerned about anxiety, depression, burnout, insomnia, and moral distress under unusual circumstances, which force wearing cumbersome and uncomfortable personal protective equipment and keeping up with emerging knowledge and new technologies of COVID-19 (10). Singh et al. (8) systematic review meta-analysis has also shown that healthcare workers experienced a crisis of psychological well-being. In addition, our study revealed that healthcare workers bear an additional burden of nursing care for elderly patients in the aging society. A recent report from the United Kingdom also shows that in the early stages of the COVID-19 pandemic, many nurses felt strong concerns about their work environment (especially in infection control), maintaining nursing quality, and maintaining mental health. It has also been found that there is inadequate access to systems to care for them (11). International surveys have also shown that health care workers feel strong psychological stress in the early stages of the COVID-19 pandemic, and this is especially true for young female health care workers (12). The content of our study is a problem not only in Japan but also in other developed countries around the world where the population is aging.

It is important to find an international solution for how to care for medical professionals under tragic situations such as a COVID-19 pandemic.

To avoid excessive load and protect healthcare workers from physical and mental stress, several countermeasures should be taken into account during difficult emerging situations such as this pandemic. First, we should consider the importance of early rehabilitation interventions. According to de Biase et al. (13) delivering rehabilitation in the same way as before the COVID-19 pandemic will neither be practical nor meet patient's needs. As mentioned, rehabilitation should be modified as a needs-based, individual approach in response to the COVID-19 pandemic. Careful training of physical therapy for COVID-19 patients is required in each hospital for COVID-19. Second, early approaches for delirium prevention and management must be adapted. The Hospital Elder Life Program provides creative approaches that comprise several intervention methods, such as providing tablet computers for remote coaching and communication by professionals, volunteers, or family members and providing sleep enhancement methods (14, 15). Early interventions for adequate programmes for COVID-19 patients, especially elderly patients, are necessary. Third, both healthcare organizations and healthcare workers should acquire knowledge or skills in stress reduction techniques. Callus et al. have summarized the key interventions for healthcare workers to enhance awareness, self-care interventions, mental health services, digital technologies, and organization approaches (16). It is important to prepare these key interventions in ordinary times because the rapid implementation of these interventions during the pandemic suggests that safety may not have received sufficient attention (17). Although the abovementioned methods will require additional work for the initial period of time, they will be of great help to maintain good conditions for both healthcare workers and hospital infrastructure in the long term.

There are several limitations in this study. This study had retrospective nature and small sample size. In addition, this study was conducted in a single medical center. These conditions can cause outcome reporting bias and patient selection bias. Because we did not evaluate the burden of nursing care quantitatively, it was difficult to explain the burden in a quantitative manner. Nonetheless, we believe that our study had a significant impact to reveal a burden of nursing care for elderly COVID-19 patients during COVID-19 pandemic.

In conclusion, we found that the proportion of elderly patients aged 80 years or older was relatively high during the hospitalization for COVID-19. Poor ADL at admission in these elderly patients was significantly associated with poor prognosis of COVID-19. We should keep in mind that healthcare workers are forced to have an additional burden of nursing care in the aging society during the emerging COVID-19 pandemic. Interventions to reduce the burden for both patients and healthcare workers are urgently needed.

## Data Availability Statement

Data are available from COVIREGI-JP bureau with permission. Only participating institutions can apply for data use.

## Ethics Statement

The studies involving human participants were reviewed and approved by NHO Kyoto Medical Center Review Boards. Written informed consent for participation was not required for this study in accordance with the national legislation and the institutional requirements (approval number: 20–092).

## Author Contributions

KF and EK drafted and revised the manuscript. KF, EK, OK, and HH collected the data for this study. HH, AY, TO, and TM supervised the study and revised the manuscript. All the authors have approved the manuscript for submission.

## Conflict of Interest

The authors declare that the research was conducted in the absence of any commercial or financial relationships that could be construed as a potential conflict of interest.

## Publisher's Note

All claims expressed in this article are solely those of the authors and do not necessarily represent those of their affiliated organizations, or those of the publisher, the editors and the reviewers. Any product that may be evaluated in this article, or claim that may be made by its manufacturer, is not guaranteed or endorsed by the publisher.
